# Association of Vitamin D Levels and Mortality in Overactive Bladder: Nonlinear Dose–Response and Threshold Effect

**DOI:** 10.1002/fsn3.71722

**Published:** 2026-04-02

**Authors:** Chao Yang, Nanshan Shen, Haitao Xiao, Zhendong Zhao, Qizhi Yang, Yifan Liu, Dehui Lai

**Affiliations:** ^1^ Department of Urology 302 Hospital of China Guizhou Aviation Industry Group Anshun Guizhou China; ^2^ Department of Urology The Fifth Affiliated Hospital of Guangzhou Medical University Guangzhou China

**Keywords:** 25‐hydroxyvitamin D, cardiovascular diseases, mortality, NHANES, overactive bladder

## Abstract

The relationship between 25‐hydroxyvitamin D [25(OH)D] and overall and cause‐specific mortality among patients with overactive bladder (OAB) remains unclear. This study aimed to clarify whether serum 25(OH)D levels were associated with all‐cause and cardiovascular disease (CVD) mortality in patients with OAB. We analyzed data from patients with OAB enrolled in the 2005–2018 National Health and Nutrition Examination Survey (NHANES). We used the Cox proportional hazards regression model to explore the associations between 25(OH)D levels and overall and CVD mortality in patients with OAB. We visualized this relationship using restricted cubic splines (RCS) and further determined the threshold using piecewise functions. Of the 4681 eligible participants, 745 overall and 219 CVD deaths occurred. Multivariate Cox regression adjustment revealed an L‐shaped relationship between serum 25(OH)D concentration and overall mortality and a U‐shaped relationship with CVD mortality. The breakpoint for all‐cause mortality was 66.82 nmol/L, and that for CVD mortality was 119.06 nmol/L. Relative to the lower group, the all‐cause mortality risk in the higher concentration group (≥ 66.82 nmol/L) decreased by 27% (HR = 0.73, 95% CI: 0.62–0.85) in patients with OAB. Among patients with OAB, serum 25(OH)D shows an inverse, non‐linear association with overall and CVD mortality, for which the thresholds for overall and CVD mortality are 66.82 and 119.06 nmol/L, respectively. Our study provides reference values for the health management of patients with OAB, but further confirmation is still required through randomized controlled trials.

## Graphical abstract

This study investigated the association between serum 25‐hydroxyvitamin D [25(OH)D] levels and mortality risk in 4681 patients with overactive bladder (OAB) from the 2005 to 2018 NHANES cohort. Restricted cubic spline analysis revealed a non‐linear dose–response relationship, namely an L‐shaped association with all‐cause mortality (threshold: 66.82 nmol/L) and a U‐shaped association with cardiovascular mortality (threshold: 119.06 nmol/L). These findings suggest that maintaining optimal 25(OH)D levels may help reduce mortality risk in patients with OAB, thus providing specific reference values for clinical management.

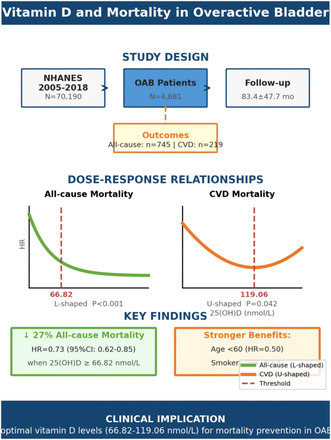

## 1 Introduction

Overactive bladder (OAB) is a common chronic disorder characterized mainly by frequent urination, urgent urination, or increased nocturia (Anis et al. 2025). The incidence rate of OAB is approximately 16%, and it tends to increase with age (Basra and Kelleher 2007; Coyne et al. 2011; Erdem and Chu 2006). OAB affects patients in multiple aspects, including their psychological well‐being, social life, and physical health. It is also associated with increased cardiovascular risk, mortality, and other complications compared to individuals without OAB (Asche et al. 2012; Gong and Huang 2024). Furthermore, some medications used to treat OAB may increase the risk of death (Welk 2023). Therefore, identifying modifiable factors to reduce mortality in patients with OAB is critical.

Serum 25‐hydroxyvitamin D [25(OH)D], comprising both D3 and D2, plays a key role in regulating calcium and phosphate homeostasis (Giustina et al. 2024). Previous studies have shown that 25(OH)D is related to the function of multiple physiological systems (Bouillon et al. 2019; Charoenngam and Holick 2020; Flexeder et al. 2017; Hung et al. 2023; Jhee et al. 2018; Sha et al. 2025). Some real‐world studies and Mendelian randomization analyses have demonstrated that insufficient 25(OH)D is closely associated with mortality in various populations, including UK Biobank participants aged 40–73 years (Sha et al. 2023), U.S. adults aged ≥ 40 years from NHANES (Liu et al. 2025), and UK Biobank individuals of White European ancestry (aged 37 to 73 years at recruitment) (Sutherland et al. 2022). However, a large‐scale Mendelian randomization study involving 500,962 individuals with no known history of coronary heart disease or stroke at baseline found no causal association between 25(OH)D and mortality (Sofianopoulou et al. 2024). There has been consistent controversy regarding the research on 25(OH)D (Gallagher and Rosen 2023). A clinical study has shown that serum 25(OH)D insufficiency is linked to a higher OAB risk (Markland et al. 2019). However, the relationship between 25(OH)D and the overall and cause‐specific mortality among OAB patients remains unknown. Therefore, establishing this association and delineating the optimal 25(OH)D threshold is imperative.

To address these knowledge gaps, we carried out a prospective cohort study utilizing the large‐scale U.S. National Health and Nutrition Examination Survey (NHANES) to investigate the relationship between 25(OH)D levels and overall and cause‐specific mortality in patients with OAB.

## 2 Methods

### 2.1 Study Design and Population

In the United States, NHANES is a nationwide health survey led by the Centers for Disease Control and Prevention (CDC) and the National Center for Health Statistics (NCHS), aiming to evaluate nutritional status and health among the American population. The research protocol is approved by the Institutional Review Board (IRB), and informed consent is obtained from all participants. This investigation adhered to the principles of the Declaration of Helsinki.

We collected data for seven cycles from 2005 to 2018. This study surveyed a total of 70,190 individuals, among whom 6750 were diagnosed with OAB. After excluding participants with 25(OH)D data deficiency (*N* = 434), baseline cancer (*N* = 966), missing medical condition data (*N* = 8), overall mortality data (*N* = 6), and missing other covariate data (*N* = 655), 4681 participants remained in the final cohort (Figure S1).

### 2.2 Serum 25(OH)D Measurement

The DiaSorin RIA kit for 25(OH)D quantification is applied for both the 2005–2006 NHANES cycles. After 2007, the 25(OH)D measurements are transformed to standardized LC–MS/MS values by the CDC for its analytical advantages. This standardization will enable a direct comparison of vitamin D data over the years in national health surveys. Thus, the regression method is applied to transform the measured 25(OH)D levels of 2005–2006 into the standardized LC–MS/MS equivalent measured value.

### 2.3 Ascertainment of Mortality Outcomes

We used a publicly available NHANES mortality file through December 31, 2019, to determine overall and specific cause mortality. The causes of disease‐related mortality are systematically coded using the 10th Revision of the International Classification of Diseases (ICD‐10).

### 2.4 Assessment of OAB

OAB is a condition characterized by urge urinary incontinence (UUI) and an increased frequency of nocturia. The status of OAB is determined based on responses to standardized questionnaire items administered to participants in the NHANES. Table S1 provides a detailed description of the criteria defining OAB. The OAB Symptom Score (OABSS) is employed to ascertain the presence of OAB in patients, with a score of 3 or higher indicating a diagnosis of OAB (Hui et al. 2024; Tang et al. 2024) (Table S2).

### 2.5 Assessment of Covariates

The study accounted for key sociodemographic factors, age (years), gender, ethnicity/race, poverty–income ratio (PIR), and educational attainment. Ethnicity/race is categorized as Mexican American, non‐Hispanic Black, non‐Hispanic White, or other. Educational attainment is stratified into < 9 years, 9–12 years, and ≥ 12 years. Body mass index (BMI; kg/m^2^) data were obtained from the examination module. In the questionnaire module, drinking is defined as consuming ≥ 12 alcoholic beverages in the past year, and smoking is defined as someone who has smoked 100 cigarettes in their lifetime. Diabetes and hypertension are recorded as yes or no based on the doctor's diagnosis history. The participants' past medical history was obtained from the medical condition questionnaire, including cancer or malignant tumors and cardiovascular disease (CVD). CVD includes congestive heart failure, angina, heart attack, and stroke.

Serum assays measure uric acid (UA), creatinine, total cholesterol (TC), and direct high‐density lipoprotein cholesterol (HDL‐C), all reported in mg/dL. Creatinine is converted into the estimated glomerular filtration rate (eGFR) using relevant formulas (Levey et al. 2009).

### 2.6 Statistical Analysis

Continuous variables were first examined for distributional form; those approximating normality were represented as mean ± standard deviation (SD), whereas skewed data were summarized as median with interquartile range (IQR). Appropriate statistical tests were applied for group comparisons: Student's *t*‐test for parametric comparisons and the Mann–Whitney *U* test otherwise, while categorical comparisons relied on the *χ* ^2^ test. We divided serum 25(OH)D levels into quartiles (Q1–Q4). We employed four Cox proportional‐hazards models to observe the association with mortality risk. Restricted cubic splines (RCS) were utilized to visualize this relationship. If the relationship was nonlinear, we further employed two‐segment Cox models to study the potential relationship. Subgroup results were stratified by age, gender, race, diabetes, hypertension, BMI, smoking, and drinking.

A sensitivity analysis was performed to evaluate the consistency and reliability of the findings, including individuals < 2 years of follow‐up, extreme values of 0.5% at both ends of 25(OH)D levels, and those with baseline CVD. Additionally, we utilized propensity score matching (PSM) statistical methods for the sensitivity analysis. All data were processed in R (v4.2.2) and Free Statistics (v2.1.1), with two‐sided *p* values < 0.05 reported as statistically significant.

## 3 Results

### 3.1 Baseline Characteristics of the Research Participants

In this study, data of 4681 patients (mean age 57.2 ± 16.4 years) were analyzed; of these 39.9% were men and 60.1% were women. During a mean follow‐up of 83.4 ± 47.7 months, 745 individuals experienced overall mortality, 219 suffered from CVD mortality, and 143 suffered from cancer mortality. Baseline demographics were grouped by serum 25(OH)D quartile (Table 1).

**TABLE 1:** Baseline characteristics of participants with OAB according to serum 25(OH)D concentrations quartiles.

Variables	Total (*N* = 4681)	Serum 25(OH)D concentrations (nmol/L)				*p*
		Q1 (6.31, 42.9)	Q2 (42.9, 60.4)	Q3 (60.4, 80)	Q4 (80, 422)	
		Q1 (*N* = 1169)	Q2 (*N* = 1170)	Q3 (*N* = 1171)	Q4 (*N* = 1171)	
Age (years)	57.2 ± 16.4	52.7 ± 16.3	55.8 ± 16.1	57.9 ± 16.2	62.5 ± 15.4	< 0.001
Gender (%)						< 0.001
Male (%)	1867 (39.9)	422 (36.1)	498 (42.6)	513 (43.8)	434 (37.1)	
Female	2814 (60.1)	747 (63.9)	672 (57.4)	658 (56.2)	737 (62.9)	
Race						< 0.001
Non‐Hispanic White (%)	1702 (36.4)	202 (17.3)	342 (29.2)	527 (45)	631 (53.9)	
Non‐Hispanic Black (%)	1394 (29.8)	614 (52.5)	328 (28)	237 (20.2)	215 (18.4)	
Mexican American (%)	761 (16.3)	191 (16.3)	253 (21.6)	192 (16.4)	125 (10.7)	
Other(%)	824 (17.6)	162 (13.9)	247 (21.1)	215 (18.4)	200 (17.1)	
Education (%)						< 0.001
< 9	745 (15.9)	160 (13.7)	235 (20.1)	204 (17.4)	146 (12.5)	
9–12	2047 (43.7)	555 (47.5)	511 (43.7)	500 (42.7)	481 (41.1)	
> 12	1889 (40.4)	454 (38.8)	424 (36.2)	467 (39.9)	544 (46.5)	
PIR	1.6 (0.9, 3.0)	1.4 (0.8, 2.5)	1.5 (0.9, 2.7)	1.7 (0.9, 3.3)	2.0 (1.1, 3.8)	< 0.001
BMI (kg/m^2^)	31.4 ± 7.9	33.6 ± 9.2	31.7 ± 7.4	30.9 ± 7.4	29.6 ± 6.7	< 0.001
Diabetes (%)						0.963
No	3540 (75.6)	883 (75.5)	890 (76.1)	887 (75.7)	880 (75.1)	
Yes	1141 (24.4)	286 (24.5)	280 (23.9)	284 (24.3)	291 (24.9)	
Hypertension (%)						< 0.001
No	2138 (45.7)	551 (47.1)	571 (48.8)	543 (46.4)	473 (40.4)	
Yes	2543 (54.3)	618 (52.9)	599 (51.2)	628 (53.6)	698 (59.6)	
Smoking (%)						0.158
No	2333 (49.8)	557 (47.6)	608 (52)	573 (48.9)	595 (50.8)	
Yes	2348 (50.2)	612 (52.4)	562 (48)	598 (51.1)	576 (49.2)	
Drinking (%)						0.201
No	1458 (31.1)	386 (33)	373 (31.9)	358 (30.6)	341 (29.1)	
Yes	3223 (68.9)	783 (67)	797 (68.1)	813 (69.4)	830 (70.9)	
TC (mg/dL)	194.2 ± 44.8	193.5 ± 45.5	195.5 ± 45.5	194.6 ± 42.9	193.2 ± 45.1	0.577
HDL‐C (mg/dL)	53.7 ± 16.7	53.0 ± 17.0	51.8 ± 15.6	53.1 ± 16.0	57.1 ± 17.5	< 0.001
UA (mg/dL)	5.5 ± 1.6	5.6 ± 1.7	5.6 ± 1.5	5.5 ± 1.5	5.5 ± 1.6	0.226
eGFR (mL/min/1.73 m^2^)	95.3 ± 25.0	98.8 ± 25.3	97.8 ± 24.2	95.0 ± 24.4	89.5 ± 25.1	< 0.001
All‐cause mortality (%)						0.002
No	3936 (84.1)	945 (80.8)	996 (85.1)	1013 (86.5)	982 (83.9)	
Yes	745 (15.9)	224 (19.2)	174 (14.9)	158 (13.5)	189 (16.1)	
CVD mortality (%)						0.061
No	4462 (95.3)	1099 (94)	1126 (96.2)	1122 (95.8)	1115 (95.2)	
Yes	219 (4.7)	70 (6)	44 (3.8)	49 (4.2)	56 (4.8)	
Cancer mortality (%)						0.105
No	4538 (96.9)	1131 (96.7)	1137 (97.2)	1145 (97.8)	1125 (96.1)	
Yes	143 (3.1)	38 (3.3)	33 (2.8)	26 (2.2)	46 (3.9)	
Follow‐up time (months)	83.4 ± 47.7	86.7 ± 48.4	88.5 ± 48.7	85.6 ± 47.8	73.0 ± 44.4	< 0.001

*Note:* Normally distributed continuous variables are presented as mean ± standard deviation (SD), and skewed variables as median (interquartile range). Categorical variables are expressed as *n* (%).

Abbreviations: BMI, body mass index; CVD, cardiovascular disease; eGFR, estimated glomerular filtration rate; HDL‐C, high‐density lipoprotein cholesterol; OAB, overactive bladder; PIR, poverty income ratio; TC, total cholesterol; UA, uric acid; 25(OH)D, 25‐hydroxyvitamin D.

### 3.2 Association Between Serum 25(OH)D Concentration and Mortality

To observe the effect of 25(OH)D on mortality, four Cox proportional‐hazards models were fitted (Table 2). The first quartile (Q1) served as the baseline category (hazard ratio [HR], 1.00; 95% confidence interval [CI], 1.00). We found that as 25(OH)D concentrations increased, overall mortality and CVD deaths among patients with OAB gradually decreased. Relative to Q1, the Q3 group (HR: 0.49, 95% CI: 0.4–0.6, *P* for trend < 0.001) had a significant 51% reduction in overall mortality risk in the fully adjusted model. Similarly, the Q4 group (HR: 0.47, 95% CI: 0.32–0.69, *P* for trend < 0.001) had a significant 53% reduction in CVD mortality risk in the fully adjusted model. However, there was no significant difference in cancer mortality.

**TABLE 2:** Multivariate Cox proportional hazards model regression analysis.

	Serum 25(OH)D concentrations (nmol/L)								*p* trend
	Q1		Q2		Q3		Q4		
	HR (95% CI)	*p*	HR (95% CI)	*p*	HR (95% CI)	*p*	HR (95% CI)	*p*	
All‐cause mortality									
Model 1	1	1 (Ref)	0.76 (0.62, 0.92)	0.006	0.72 (0.58, 0.88)	0.001	1.04 (0.85, 1.26)	0.724	0.925
Model 2	1	1 (Ref)	0.61 (0.5, 0.75)	< 0.001	0.49 (0.39, 0.6)	< 0.001	0.56 (0.46, 0.69)	< 0.001	< 0.001
Model 3	1	1 (Ref)	0.62 (0.51, 0.76)	< 0.001	0.49 (0.39, 0.6)	< 0.001	0.55 (0.45, 0.68)	< 0.001	< 0.001
Model 4	1	1 (Ref)	0.63 (0.52, 0.77)	< 0.001	0.49 (0.4, 0.6)	< 0.001	0.54 (0.44, 0.67)	< 0.001	< 0.001
CVD mortality									
Model 1	1	1 (Ref)	0.62 (0.42, 0.9)	0.012	0.71 (0.49, 1.02)	0.066	0.96 (0.68, 1.37)	0.821	0.837
Model 2	1	1 (Ref)	0.5 (0.34, 0.74)	< 0.001	0.47 (0.32, 0.69)	< 0.001	0.48 (0.33, 0.7)	< 0.001	< 0.001
Model 3	1	1 (Ref)	0.51 (0.35, 0.75)	0.001	0.47 (0.32, 0.69)	< 0.001	0.49 (0.33, 0.71)	< 0.001	< 0.001
Model 4	1	1 (Ref)	0.51 (0.35, 0.76)	0.001	0.48 (0.33, 0.7)	< 0.001	0.47 (0.32, 0.69)	< 0.001	< 0.001
Cancer mortality									
Model 1	1	1 (Ref)	0.85 (0.53, 1.36)	0.495	0.7 (0.42, 1.15)	0.156	1.49 (0.97, 2.3)	0.068	0.131
Model 2	1	1 (Ref)	0.74 (0.46, 1.19)	0.219	0.55 (0.33, 0.92)	0.023	1.02 (0.64, 1.63)	0.942	0.919
Model 3	1	1 (Ref)	0.77 (0.48, 1.23)	0.275	0.56 (0.33, 0.94)	0.03	1.05 (0.65, 1.68)	0.847	0.987
Model 4	1	1 (Ref)	0.76 (0.47, 1.22)	0.257	0.56 (0.33, 0.94)	0.028	1.03 (0.64, 1.65)	0.907	0.934

*Note:* Model 1: Non‐adjusted. Model 2: Adjusted for age, gender, race, education, PIR. Model 3: Adjusted for age, gender, race, education, PIR, BMI, diabetes, hypertension, smoking, drinking. Model 3: Adjusted for age, gender, race, education, PIR, BMI, diabetes, hypertension, smoking, drinking, TC, direct HDL‐C, UA, eGFR.

**FIGURE 1:**
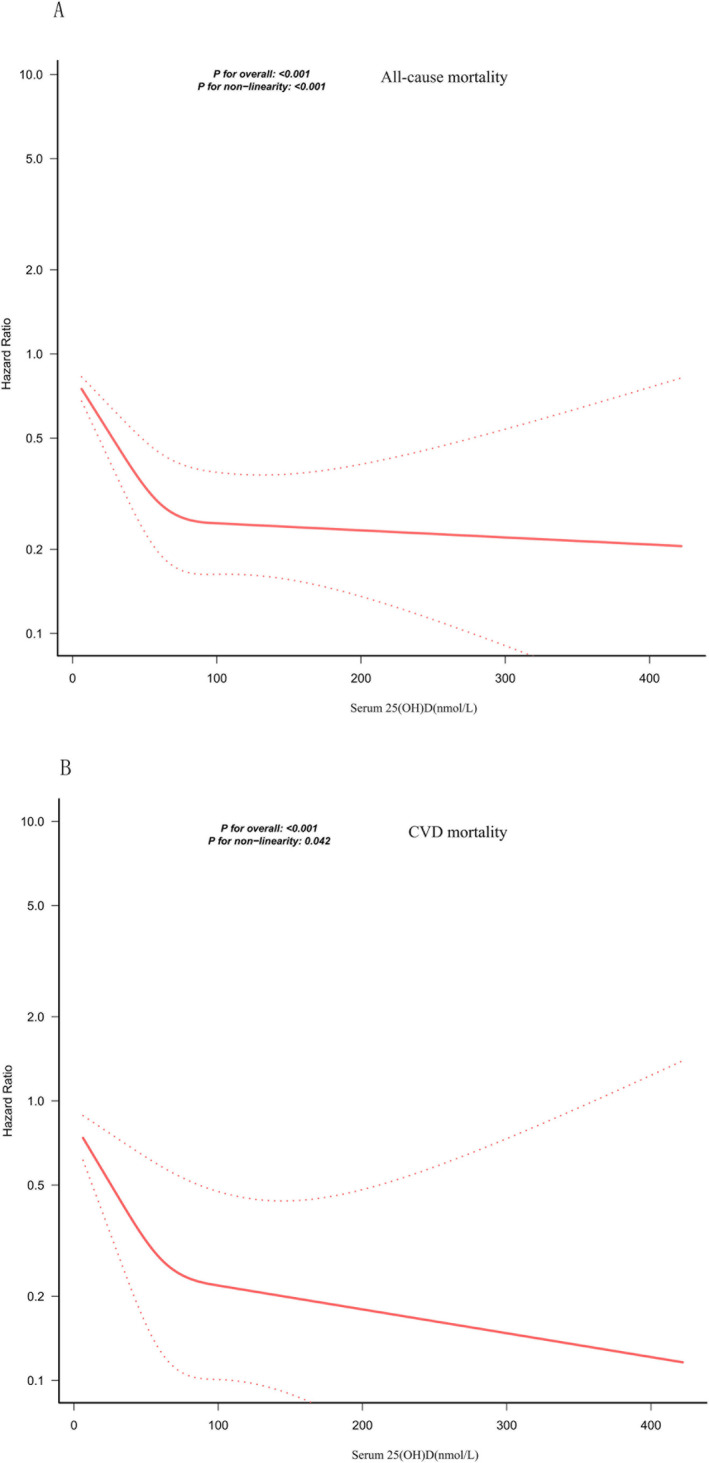
Association between 25(OH)D concentration and all‐cause mortality (A) and CVD mortality (B) in patients with OAB (Adjusted for age, gender, race, education, PIR, BMI, diabetes, hypertension, smoking, drinking, TC, direct HDL‐C, UC, eGFR. The solid and dotted lines represent the estimated HR values and their corresponding 95% CIs, respectively).

We plotted RCS to explore the potential non‐linear associations between 25(OH)D levels and mortality. A multivariate‐adjusted RCS analysis demonstrated significant non‐linearity for overall mortality (*P*_non‐linearity < 0.001) and CVD mortality (*P*_non‐linearity = 0.042). The curve for overall mortality displayed an L‐shaped pattern, whereas the CVD mortality curve followed a U‐shaped trajectory (Figure 1).

We employed the Cox model to further explore the piecewise non‐linear relationship. We found that the breakpoints for overall and CVD mortality were 66.82 and 119.06 nmol/L, respectively. Below these cut‐offs, each 10 nmol/L decrement in 25(OH)D corresponded to an 18% rise in adjusted overall mortality risk (HR: 0.982, 95% CI: 0.975–0.988) and an 11% rise in CVD mortality (HR: 0.989, 95% CI: 0.982–0.995). Above 66.82 nmol/L, the overall mortality risk plateaued, indicating a threshold saturation effect (HR: 1.005, 95% CI: 0.995–1.007; *p* = 0.8603). Conversely, when concentrations exceeded 119.06 nmol/L, the difference in CVD mortality risk was not significant; however, it suggested that excessively high 25(OH)D may pose a potential CVD mortality risk (HR: 1.163; 95% CI: 0.955, 1.417; *p* = 0.132) (Table 3).

**TABLE 3:** Threshold effect analysis of serum 25(OH)D concentrations on all‐cause and CVD mortality in OAB patients.

	Adjusted HR (95% CI)	*p*
All‐cause mortality		
Breakpoint	66.82	
25(OH)D concentrations < 66.82 nmol/L	0.982 (0.975, 0.988)	< 0.001
25(OH)D concentrations ≥ 66.82 nmol/L	1.0005 (0.9945, 1.0066)	0.8603
Likelihood Ratio test		< 0.001
CVD mortality		
Breakpoint	119.06	
25(OH)D concentrations < 119.06 nmol/L	0.989 (0.982, 0.995)	< 0.001
25(OH)D concentrations ≥ 119.06 nmol/L	1.163 (0.955, 1.417)	0.1323
Likelihood Ratio test		0.003

*Note:* Adjusted for age, gender, race, education, PIR, BMI, diabetes, hypertension, smoking, drinking, TC, direct HDL‐C, UA, eGFR.

### 3.3 Subgroup Analysis

**FIGURE 2:**
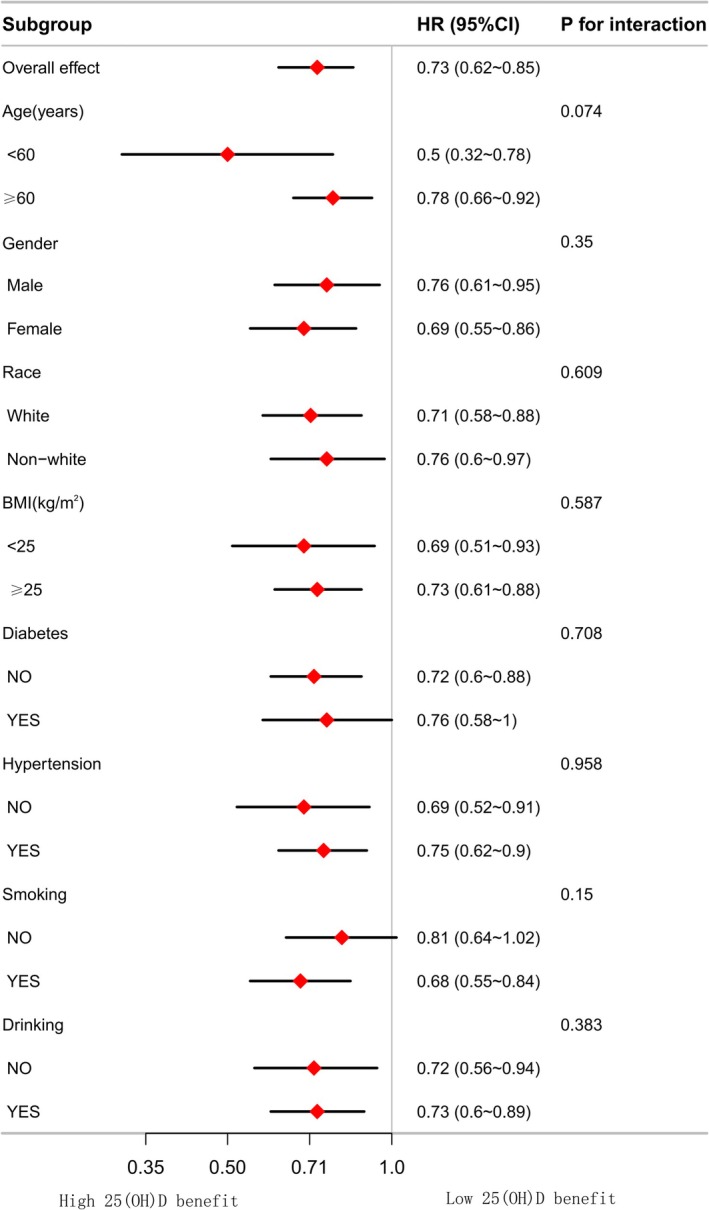
Forest plots of subgroup analysis for the association between 25(OH)D and all‐cause mortality (apart from the subgroup variable itself, all other covariates have been adjusted).

We further analyzed the effects of different 25(OH)D levels in each subgroup population. Across all strata, serum 25(OH)D ≥ 66.82 nmol/L was linked to a significant 27% lower risk of overall mortality (HR: 0.73, 95% CI: 0.62–0.85). This protective effect was consistent across strata defined by age, gender, race, hypertension, diabetes, BMI, smoking, and drinking, with no significant interaction detected between the subgroups. Although the interaction tests did not reach statistical significance, the most pronounced risk reductions were observed in individuals aged < 60 years (HR: 0.50, 95% CI: 0.32–0.78; *P*_interaction = 0.074) and smokers (HR: 0.68, 95% CI: 0.55–0.84; *P*_interaction = 0.15) (Figure 2).

### 3.4 Sensitivity Analysis

To assess the stability of our research conclusions, we conducted a sensitivity analysis (Table S3). After excluding cases with follow‐up times of less than 2 years (*N* = 513), and 0.5% of the extreme values of vitamin D in each group (*N* = 47), and those with CVD at baseline (*N* = 930), the inverse relationship between 25(OH)D and overall mortality was still consistent in all adjusted models. After conducting PSM, the 1577 pairs in each group were well matched, with no statistically significant differences between the two groups (Figure S2). The HR for overall mortality was 0.74 (95% CI: 0.63–0.87, *p* < 0.01) after PSM. The above sensitivity analysis results indicate that our research conclusions are robust again.

## 4 Discussion

In this extensive prospective cohort, higher serum 25(OH)D levels were linked to lower overall and CVD mortality risk in patients with OAB. Serum 25(OH)D demonstrated L‐shaped associations with overall mortality, whereas its link with CVD mortality followed a U‐shaped pattern. The optimal cut‐off point for serum 25(OH)D concentrations for overall mortality was 66.82 nmol/L. Above this concentration, there is no significant benefit (HR = 1.0005, *p* = 0.86). However, for cardiovascular mortality, the cut‐off point was even higher at 119.06 nmol/L. After exceeding this point, there may be potential mortality risks (HR = 1.16, *p* = 0.13). Furthermore, we found that the negative link between higher 25(OH)D levels and overall mortality was more strongly influenced by age and smoking.

Although OAB itself is rarely fatal, a growing body of evidence suggests that it is closely associated with conditions that significantly increase mortality risk, particularly CVD and metabolic syndrome (Asche et al. 2012; Gong and Huang 2024). With the acceleration of the global aging population, the number of individuals with OAB is expected to increase significantly (Basra and Kelleher 2007; Coyne et al. 2011; Erdem and Chu 2006). Anticholinergic drugs and β3 agonists are the main clinical medications used to treat OAB (Welk 2023). The use of these drugs has been proven to be a strong independent risk factor for all‐cause, CVD, and cancer mortality (Wei et al. 2026; Welk 2023). The higher mortality risk of patients with OAB compared to that of the general population may be related to specific pathophysiological mechanisms. First, chronic low‐grade inflammation is believed to play a role in the pathogenesis of OAB and is also a well‐established driver of atherosclerosis and CVD (Blondon et al. 2016; Zúñiga and Bazan‐Perkins 2025). Second, autonomic nervous system dysfunction not only plays a significant role in OAB (Aydogmus et al. 2017) but also modulates cardiac function and vascular tone through its sympathetic and parasympathetic branches (Ziegler et al. 2025). Third, OAB and CVD share numerous common risk factors, including aging, obesity, smoking, and diabetes mellitus. Given these pathological and physiological mechanisms of OAB, it is particularly important to study the risks associated with this specific group of people with OAB.

Whether for the general population or specific groups, the majority of investigations report that insufficient 25(OH)D is closely associated with overall and CVD mortality (Sutherland et al. 2022; Wan et al. 2021; Xiao et al. 2022; Yang et al. 2023). The Endocrine Society recommends maintaining 25(OH)D levels at ≥ 50 nmol/L to support optimal health. A study using data from the NHANES collected in 2556 individuals with osteoarthritis indicated that 25(OH)D levels were associated with overall and CVD mortality. The 25(OH)D threshold values were 27.70 and 54.40 nmol/L, respectively (Xiao et al. 2022). Another prospective study involving 18,797 people from the Korean population concluded that maintaining 25(OH)D levels at approximately 50–60 nmol/L might help extend lifespan (Song et al. 2024). These research findings are consistent with our conclusions. However, in our study, for patients with OAB, the optimal 25(OH)D levels for reducing overall and CVD mortality were 66.82 and 119.06 nmol/L, respectively. These values are higher than those reported in other studies. This may be related to the specific pathophysiology of patients with OAB (Peyronnet et al. 2019). The chronic inflammatory state associated with OAB leads to increased demand for 25(OH)D (Blondon et al. 2016; Zúñiga and Bazan‐Perkins 2025). Furthermore, it may be associated with decreased expression of vitamin D receptors caused by chronic inflammation (Bakke and Sun 2018; Zhang et al. 2021). The U‐shaped curve in this study indicates that patients with OAB need to avoid excessively high serum vitamin D levels, especially when they have CVD. It is particularly crucial to investigate the optimal 25(OH)D levels for specific diseases to reduce mortality (Grant et al. 2022). Interestingly, from the group analysis, we also found that for people under the age of 60 years, the inverse associations between 25(OH)D levels and mortality risk were more significant. Contrary to prior studies, this may be related to the specific OAB group.

25(OH)D reduces mortality through the following mechanisms. It improves cardiovascular function by modulating renin‐angiotensin‐aldosterone system (RAAS) activity and vascular endothelial function, among other pathways (de la Guía‐Galipienso et al. 2021; Lee et al. 2008; Renke et al. 2023). In the sensitivity analysis of this study, when patients with CVD at baseline were excluded, higher levels of 25(OH)D did not significantly improve the risk of overall mortality. These theoretical foundations and research findings confirm that 25(OH)D exerts cardioprotective effects, lowering both overall and cardiovascular mortality through immune regulation (Suaini et al. 2015). Our research revealed that among smokers, individuals with higher concentrations of 25(OH)D experienced more significant benefits. Smoking can lead to chronic inflammation and the occurrence of various malignant tumors. 25(OH)D reduces the occurrence of malignant tumors and improves the prognosis of chronic diseases by enhancing the immune system, thereby reducing the risk of mortality (Færk et al. 2018; Sun et al. 2018). 25(OH)D may also reduce the occurrence of metabolic diseases, including hyperlipidemia and diabetes, by regulating protein pathways (Aatsinki et al. 2019; Asano et al. 2017). In conclusion, the mechanism by which 25(OH)D reduces overall and CVD mortality is intricate and warrants further investigation for full elucidation.

Due to the insufficiency of sunlight‐induced vitamin D synthesis to meet the body's requirements, individuals often need to supplement vitamin D through the biofortification of common foods or by taking medications (Neill et al. 2023). For instance, foods rich in vitamin D, such as eggs, milk, and fish, have become important sources for supplementing vitamin D (Guo et al. 2018). The risk of developing osteoporosis is higher in people aged > 50 years, especially in women (Morin et al. 2025). Consequently, they may focus more on their health and blindly supplement vitamin D. Excessive vitamin D levels in the body not only increase toxicity but also induce hypercalciuria, which increases the risk of OAB (Marcinowska‐Suchowierska et al. 2018; Matsuo et al. 2022), especially for women during the perimenopausal period (Zhang et al. 2025). Therefore, when consuming daily foods, we should focus more on the health of women's bladders.

Our research presents several notable advantages. Firstly, it used the NHANES cohort's extensive, large, nationally representative sample and prospective design to rigorously assess the associations between serum 25(OH)D levels and mortality risk among patients with OAB, while also establishing an optimal 25(OH)D threshold for these associations. This study is the first to determine such an optimal threshold. Secondly, our research incorporated a diverse array of covariates, which can enhance the validity of our conclusions by adjusting for multiple potential confounding factors. Lastly, in addition to employing conventional multiple regression and subgroup analysis, this study conducted a sensitivity analysis and comprehensively applied PSM to control for confounding factors. Consequently, the conclusions drawn are more robust and reliable.

However, our research also has some limitations. First, this is an observational study and cannot establish a causal relationship between 25(OH)D levels and mortality risk. Further verification is needed through a randomized controlled trial. Second, a single serum 25(OH)D measurement was used to assess long‐term exposure status, which might underestimate the impact of fluctuations within a certain period on the results. Future studies should repeat the detection of 25(OH)D levels to verify stability. Third, although various methods were employed to control confounding factors, unmeasured variables (such as physical activity levels, duration of sunlight exposure, and vitamin D supplement dosage) may still affect the results. In future research, we will integrate multidimensional data to enhance accuracy. Finally, the diagnosis of OAB in this study was based on self‐reported questionnaire responses and the OAB Symptom Score. Although this method is widely used in the NHANES database, it lacks clinical validation via more objective assessments, such as urodynamic studies or detailed voiding diaries. This factor may introduce potential misclassification bias and should be considered when interpreting our findings.

## 5 Conclusion

This investigation demonstrated a significant, non‐linear association between serum 25(OH)D levels and both overall and cardiovascular mortality in patients with OAB. We further discovered that 25(OH)D concentrations were associated with an L‐shaped pattern of overall mortality and a U‐shaped pattern of CVD mortality. The optimal threshold for 25(OH)D regarding overall mortality was 66.82 nmol/L, and the threshold for CVD mortality was 119.06 nmol/L. The subgroup with higher 25(OH)D concentrations, especially those under the age of 60 years and the smoking group, showed more significant benefits in reducing mortality risk. These results may provide a reference value for evaluating the role of 25(OH)D in preventing overall and CVD mortality in patients with OAB.

## Author Contributions

**Haitao Xiao:** methodology, software, visualization, investigation. **Zhendong Zhao:** software, data curation, formal analysis. **Yifan Liu:** funding acquisition, project administration, writing – review and editing, resources. **Nanshan Shen:** conceptualization, validation, supervision, resources, writing – original draft. **Qizhi Yang:** supervision, writing – review and editing, project administration, writing – original draft. **Chao Yang:** conceptualization, methodology, software, data curation, writing – original draft, investigation, project administration, writing – review and editing.

## Funding

This study was supported by grants from Medical Health Science and Technology Project of Guizhou Provincial Health Commission: gzwkj2023‐376, gzwkj2023‐378; Guizhou Provincial Basic Research Program (Natural Science): QianKeHe Basic‐ZK[2023]General 472; Guangzhou Major Medical Disciplines Project (2025–2027), Guang Dong Engineering Technology Research Center of Biological Targeting Diagnosis, Therapy and Rchabilitation, The Fifth Affiliated Hospital of Guangzhou Medical University.

## Ethics Statement

The NHANES is a public database. The NHANES was authorized by the National Center for Health Statistics (NCHS) Ethics Review Committee. This study was conducted in accordance with the Declaration of Helsinki.

## Consent

All participants completed written informed consent forms before participation.

## Conflicts of Interest

The authors declare no conflicts of interest.

## Supporting information


**Figure S1:** Flowchart of participant screening.

**Figure S2:** Propensity score matching in two groups of vitamin D concentrations.

**Table S1:** Detailed description of the relevant variables of OAB in the NHANES database.
**Table S2:** OAB symptom score from NHANES.
**Table S3:** Sensitivity analysis.

## Data Availability

Publicly available datasets were analyzed in this study. This data can be found here: https://www.cdc.gov/nchs/nhanes/.

## References

[fsn371722-bib-0001] Aatsinki, S. M. , M. S. Elkhwanky , O. Kummu , et al. 2019. “Fasting‐Induced Transcription Factors Repress Vitamin D Bioactivation, a Mechanism for Vitamin D Deficiency in Diabetes.” Diabetes 68, no. 5: 918–931. 10.2337/db18-1050. 30833469 PMC6477896

[fsn371722-bib-0002] Anis, O. , S. H. Jeong , and S. P. Vasavada . 2025. “Clinical Consultation Guide: A Patient‐Tailored Approach to Managing Overactive Bladder in Nursing Home Residents.” European Urology Focus 11, no. 2: 195–197. 10.1016/j.euf.2025.03.008. 40118691

[fsn371722-bib-0003] Asano, L. , M. Watanabe , Y. Ryoden , et al. 2017. “Vitamin D Metabolite, 25‐Hydroxyvitamin D, Regulates Lipid Metabolism by Inducing Degradation of SREBP/SCAP.” Cell Chemical Biology 24, no. 2: 207–217. 10.1016/j.chembiol.2016.12.017. 28132894

[fsn371722-bib-0004] Asche, C. V. , J. Kim , A. S. Kulkarni , P. Chakravarti , and K. E. Andersson . 2012. “Presence of Central Nervous System, Cardiovascular and Overall Co‐Morbidity Burden in Patients With Overactive Bladder Disorder in a Real‐World Setting.” BJU International 109, no. 4: 572–580. 10.1111/j.1464-410X.2011.10436.x. 21777361

[fsn371722-bib-0005] Aydogmus, Y. , S. Uzun , F. C. Gundogan , U. H. Ulas , T. Ebiloglu , and M. T. Goktas . 2017. “Is Overactive Bladder a Nervous or Bladder Disorder? Autonomic Imaging in Patients With Overactive Bladder via Dynamic Pupillometry.” World Journal of Urology 35, no. 3: 467–472. 10.1007/s00345-016-1880-9. 27311587

[fsn371722-bib-0006] Bakke, D. , and J. Sun . 2018. “Ancient Nuclear Receptor VDR With New Functions: Microbiome and Inflammation.” Inflammatory Bowel Diseases 24, no. 6: 1149–1154. 10.1093/ibd/izy092. 29718408 PMC6148749

[fsn371722-bib-0007] Basra, R. , and C. Kelleher . 2007. “Disease Burden of Overactive Bladder: Quality‐Of‐Life Data Assessed Using ICI‐Recommended Instruments.” PharmacoEconomics 25, no. 2: 129–142. 10.2165/00019053-200725020-00005. 17249855

[fsn371722-bib-0008] Blondon, M. , M. Cushman , N. Jenny , et al. 2016. “Associations of Serum 25‐Hydroxyvitamin D With Hemostatic and Inflammatory Biomarkers in the Multi‐Ethnic Study of Atherosclerosis.” Journal of Clinical Endocrinology and Metabolism 101, no. 6: 2348–2357. 10.1210/jc.2016-1368. 27023449 PMC4891795

[fsn371722-bib-0009] Bouillon, R. , C. Marcocci , G. Carmeliet , et al. 2019. “Skeletal and Extraskeletal Actions of Vitamin D: Current Evidence and Outstanding Questions.” Endocrine Reviews 40, no. 4: 1109–1151. 10.1210/er.2018-00126. 30321335 PMC6626501

[fsn371722-bib-0010] Charoenngam, N. , and M. F. Holick . 2020. “Immunologic Effects of Vitamin D on Human Health and Disease.” Nutrients 12, no. 7: 2097. 10.3390/nu12072097. 32679784 PMC7400911

[fsn371722-bib-0011] Coyne, K. S. , C. C. Sexton , Z. S. Kopp , C. Ebel‐Bitoun , I. Milsom , and C. Chapple . 2011. “The Impact of Overactive Bladder on Mental Health, Work Productivity and Health‐Related Quality of Life in the UK and Sweden: Results From EpiLUTS.” BJU International 108, no. 9: 1459–1471. 10.1111/j.1464-410X.2010.10013.x. 21371240

[fsn371722-bib-0012] de la Guía‐Galipienso, F. , M. Martínez‐Ferran , N. Vallecillo , C. J. Lavie , F. Sanchis‐Gomar , and H. Pareja‐Galeano . 2021. “Vitamin D and Cardiovascular Health.” Clinical Nutrition 40, no. 5: 2946–2957. 10.1016/j.clnu.2020.12.025. 33397599 PMC7770490

[fsn371722-bib-0013] Erdem, N. , and F. M. Chu . 2006. “Management of Overactive Bladder and Urge Urinary Incontinence in the Elderly Patient.” American Journal of Medicine 119, no. 3: 29–36. 10.1016/j.amjmed.2005.12.014. 16483866

[fsn371722-bib-0014] Færk, G. , Y. Çolak , S. Afzal , and B. G. Nordestgaard . 2018. “Low Concentrations of 25‐Hydroxyvitamin D and Long‐Term Prognosis of COPD: A Prospective Cohort Study.” European Journal of Epidemiology 33, no. 6: 567–577. 10.1007/s10654-018-0393-9. 29691706

[fsn371722-bib-0015] Flexeder, C. , E. Thiering , S. Koletzko , et al. 2017. “Higher Serum 25(OH)D Concentrations Are Associated With Improved FEV(1) and FVC in Adolescence.” European Respiratory Journal 49, no. 4: 1601804. 10.1183/13993003.01804-2016. 28446555

[fsn371722-bib-0016] Gallagher, J. C. , and C. J. Rosen . 2023. “Vitamin D: 100 Years of Discoveries, Yet Controversy Continues.” Lancet Diabetes and Endocrinology 11, no. 5: 362–374. 10.1016/s2213-8587(23)00060-8. 37004709

[fsn371722-bib-0017] Giustina, A. , J. P. Bilezikian , R. A. Adler , et al. 2024. “Consensus Statement on Vitamin D Status Assessment and Supplementation: Whys, Whens, and Hows.” Endocrine Reviews 45, no. 5: 625–654. 10.1210/endrev/bnae009. 38676447 PMC11405507

[fsn371722-bib-0018] Gong, H. , and S. Huang . 2024. “Associations of Overactive Bladder (OAB) With Suicidal Ideation Incidence and All‐Cause Mortality Among the U.S. Population.” BMC Psychiatry 24, no. 1: 641. 10.1186/s12888-024-06107-1. 39350063 PMC11443948

[fsn371722-bib-0019] Grant, W. B. , F. Al Anouti , B. J. Boucher , et al. 2022. “A Narrative Review of the Evidence for Variations in Serum 25‐Hydroxyvitamin D Concentration Thresholds for Optimal Health.” Nutrients 14, no. 3: 639. 10.3390/nu14030639. 35276999 PMC8838864

[fsn371722-bib-0020] Guo, J. , J. A. Lovegrove , and D. I. Givens . 2018. “25(OH)D3‐Enriched or Fortified Foods Are More Efficient at Tackling Inadequate Vitamin D Status Than Vitamin D3.” Proceedings of the Nutrition Society 77, no. 3: 282–291. 10.1017/s0029665117004062. 29173203 PMC6088524

[fsn371722-bib-0021] Hui, Z. , Z. Zewu , L. Yang , and C. Yu . 2024. “Association Between Weight‐Adjusted Waist Index and Overactive Bladder: A Cross‐Sectional Study Based on 2009‐2018 NHANES.” Frontiers in Nutrition 11: 1423148. 10.3389/fnut.2024.1423148. 39296511 PMC11408301

[fsn371722-bib-0022] Hung, M. , W. C. Birmingham , M. Ocampo , and A. Mohajeri . 2023. “The Role of Vitamin D in Cardiovascular Diseases.” Nutrients 15, no. 16: 3547. 10.3390/nu15163547. 37630735 PMC10459780

[fsn371722-bib-0023] Jhee, J. H. , K. H. Nam , S. Y. An , et al. 2018. “Severe Vitamin D Deficiency Is a Risk Factor for Renal Hyperfiltration.” American Journal of Clinical Nutrition 108, no. 6: 1342–1351. 10.1093/ajcn/nqy194. 30541088

[fsn371722-bib-0024] Lee, J. H. , J. H. O'Keefe , D. Bell , D. D. Hensrud , and M. F. Holick . 2008. “Vitamin D Deficiency an Important, Common, and Easily Treatable Cardiovascular Risk Factor?” Journal of the American College of Cardiology 52, no. 24: 1949–1956. 10.1016/j.jacc.2008.08.050. 19055985

[fsn371722-bib-0025] Levey, A. S. , L. A. Stevens , C. H. Schmid , et al. 2009. “A New Equation to Estimate Glomerular Filtration Rate.” Annals of Internal Medicine 150, no. 9: 604–612. 10.7326/0003-4819-150-9-200905050-00006. 19414839 PMC2763564

[fsn371722-bib-0026] Liu, C. , H. Wongsonegoro , T. Sheng , H. Fan , and J. Zhang . 2025. “Associations Between Serum Micronutrients and All‐Cause, Cancer, and Cardiovascular Mortality in a National Representative Population: Mediated by Inflammatory Biomarkers.” Redox Biology 81: 103573. 10.1016/j.redox.2025.103573. 40023976 PMC11915157

[fsn371722-bib-0027] Marcinowska‐Suchowierska, E. , M. Kupisz‐Urbańska , J. Łukaszkiewicz , P. Płudowski , and G. Jones . 2018. “Vitamin D Toxicity‐A Clinical Perspective.” Frontiers in Endocrinology 9: 550. 10.3389/fendo.2018.00550. 30294301 PMC6158375

[fsn371722-bib-0028] Markland, A. D. , V. Tangpricha , T. Mark Beasley , et al. 2019. “Comparing Vitamin D Supplementation Versus Placebo for Urgency Urinary Incontinence: A Pilot Study.” Journal of the American Geriatrics Society 67, no. 3: 570–575. 10.1111/jgs.15711. 30578542 PMC6403014

[fsn371722-bib-0029] Matsuo, T. , H. Ito , K. Mitsunari , K. Ohba , and Y. Miyata . 2022. “Relationship Between Urinary Calcium Excretion and Lower Urinary Tract Symptoms.” Metabolites 12, no. 3: 229. 10.3390/metabo12030229. 35323672 PMC8953485

[fsn371722-bib-0030] Morin, S. N. , W. D. Leslie , and J. T. Schousboe . 2025. “Osteoporosis: A Review.” JAMA 334, no. 10: 894–907. 10.1001/jama.2025.6003. 40587168

[fsn371722-bib-0031] Neill, H. R. , C. I. R. Gill , E. J. McDonald , W. C. McRoberts , and L. K. Pourshahidi . 2023. “The Future Is Bright: Biofortification of Common Foods Can Improve Vitamin D Status.” Critical Reviews in Food Science and Nutrition 63, no. 4: 505–521. 10.1080/10408398.2021.1950609. 34291674

[fsn371722-bib-0032] Peyronnet, B. , E. Mironska , C. Chapple , et al. 2019. “A Comprehensive Review of Overactive Bladder Pathophysiology: On the Way to Tailored Treatment.” European Urology 75, no. 6: 988–1000. 10.1016/j.eururo.2019.02.038. 30922690

[fsn371722-bib-0033] Renke, G. , B. Starling‐Soares , T. Baesso , R. Petronio , D. Aguiar , and R. Paes . 2023. “Effects of Vitamin D on Cardiovascular Risk and Oxidative Stress.” Nutrients 15, no. 3: 769. 10.3390/nu15030769. 36771474 PMC9920542

[fsn371722-bib-0034] Sha, S. , T. M. N. Nguyen , S. Kuznia , et al. 2023. “Real‐World Evidence for the Effectiveness of Vitamin D Supplementation in Reduction of Total and Cause‐Specific Mortality.” Journal of Internal Medicine 293, no. 3: 384–397. 10.1111/joim.13578. 36208176

[fsn371722-bib-0035] Sha, S. , R. Xie , T. Gwenzi , Y. Wang , H. Brenner , and B. Schöttker . 2025. “Real‐World Evidence for an Association of Vitamin D Supplementation With Atherosclerotic Cardiovascular Disease in the UK Biobank.” Clinical Nutrition 49: 118–127. 10.1016/j.clnu.2025.04.017. 40267517

[fsn371722-bib-0036] Sofianopoulou, E. , S. K. Kaptoge , S. Afzal , et al. 2024. “Estimating Dose‐Response Relationships for Vitamin D With Coronary Heart Disease, Stroke, and All‐Cause Mortality: Observational and Mendelian Randomisation Analyses.” Lancet Diabetes and Endocrinology 12, no. 1: e2–e11. 10.1016/S2213-8587(23)00287-5. 38048800 PMC7615586

[fsn371722-bib-0037] Song, S. , J. Lyu , B. M. Song , J. Y. Lim , and H. Y. Park . 2024. “Serum 25‐Hydroxyvitamin D Levels and Risk of All‐Cause and Cause‐Specific Mortality: A 14‐Year Prospective Cohort Study.” Clinical Nutrition 43, no. 9: 2156–2163. 10.1016/j.clnu.2024.07.049. 39142109

[fsn371722-bib-0038] Suaini, N. H. , Y. Zhang , P. J. Vuillermin , K. J. Allen , and L. C. Harrison . 2015. “Immune Modulation by Vitamin D and Its Relevance to Food Allergy.” Nutrients 7, no. 8: 6088–6108. 10.3390/nu7085271. 26225992 PMC4555110

[fsn371722-bib-0039] Sun, Y. Q. , B. M. Brumpton , C. Bonilla , et al. 2018. “Serum 25‐Hydroxyvitamin D Levels and Risk of Lung Cancer and Histologic Types: A Mendelian Randomisation Analysis of the HUNT Study.” European Respiratory Journal 51, no. 6: 1800329. 10.1183/13993003.00329-2018. 29748306 PMC7614587

[fsn371722-bib-0040] Sutherland, J. P. , A. Zhou , and E. Hyppönen . 2022. “Vitamin D Deficiency Increases Mortality Risk in the UK Biobank: A Nonlinear Mendelian Randomization Study.” Annals of Internal Medicine 175, no. 11: 1552–1559. 10.7326/m21-3324. 36279545

[fsn371722-bib-0041] Tang, F. , J. Zhang , R. Huang , et al. 2024. “The Association Between Wet Overactive Bladder and Consumption of Tea, Coffee, and Caffeine: Results From 2005‐2018 National Health and Nutrition Examination Survey.” Clinical Nutrition 43, no. 6: 1261–1269. 10.1016/j.clnu.2024.03.027. 38653009

[fsn371722-bib-0042] Wan, Z. , J. Guo , A. Pan , C. Chen , L. Liu , and G. Liu . 2021. “Association of Serum 25‐Hydroxyvitamin D Concentrations With All‐Cause and Cause‐Specific Mortality Among Individuals With Diabetes.” Diabetes Care 44, no. 2: 350–357. 10.2337/dc20-1485. 33168652

[fsn371722-bib-0043] Wei, K. , S. Sun , C. Chen , et al. 2026. “Thirty‐Two‐Year Trends in Anticholinergic Burden and Associated Mortality Among US Older Adults: A Population‐Based Study.” Age and Ageing 55, no. 2: afag049. 10.1093/ageing/afag049. 41785066

[fsn371722-bib-0044] Welk, B. 2023. “The Differential Risk of Mortality Among Users of Overactive Bladder Anticholinergic Medications and β3 Agonists.” European Urology Focus 9, no. 1: 168–171. 10.1016/j.euf.2022.08.002. 35987891

[fsn371722-bib-0045] Xiao, Q. , B. Cai , A. Yin , et al. 2022. “L‐Shaped Association of Serum 25‐Hydroxyvitamin D Concentrations With Cardiovascular and All‐Cause Mortality in Individuals With Osteoarthritis: Results From the NHANES Database Prospective Cohort Study.” BMC Medicine 20, no. 1: 308. 10.1186/s12916-022-02510-1. 36127705 PMC9490951

[fsn371722-bib-0046] Yang, J. , J. Lu , J. Miao , et al. 2023. “Development and Validation of a Blood Biomarker Score for Predicting Mortality Risk in the General Population.” Journal of Translational Medicine 21, no. 1: 471. 10.1186/s12967-023-04334-w. 37454089 PMC10349520

[fsn371722-bib-0047] Zhang, J. , Y. Zhang , Y. Xia , and J. Sun . 2021. “Imbalance of the Intestinal Virome and Altered Viral‐Bacterial Interactions Caused by a Conditional Deletion of the Vitamin D Receptor.” Gut Microbes 13, no. 1: 1957408. 10.1080/19490976.2021.1957408. 34375154 PMC8366551

[fsn371722-bib-0048] Zhang, Y. , F. Zou , L. Liang , et al. 2025. “Suoquan Wan Mitigates Bladder Overactivity via Modulation of Neuroimmune Homeostasis in Perimenopausal Rats.” Phytomedicine 139: 156450. 10.1016/j.phymed.2025.156450. 39922151

[fsn371722-bib-0049] Ziegler, K. A. , S. Engelhardt , D. Carnevale , et al. 2025. “Neural Mechanisms in Cardiovascular Health and Disease.” Circulation Research 136, no. 11: 1233–1261. 10.1161/circresaha.125.325580. 40403111

[fsn371722-bib-0050] Zúñiga, V. A. , and B. Bazan‐Perkins . 2025. “The Impact of Vitamin D on Atopic Disorders: Assessing Evidence for a Causal Relationship.” Frontiers in Nutrition 12: 1584818. 10.3389/fnut.2025.1584818. 40276536 PMC12018226

