# Supplementary figures and images for: Association of Vitamin D Levels and Mortality in Overactive Bladder: Nonlinear Dose–Response and Threshold Effect
Source: Food Sci Nutr. 2026 Apr 2;14(4):e71722. doi: 10.1002/fsn3.71722 (PMC13045321; doi:10.1002/fsn3.71722)

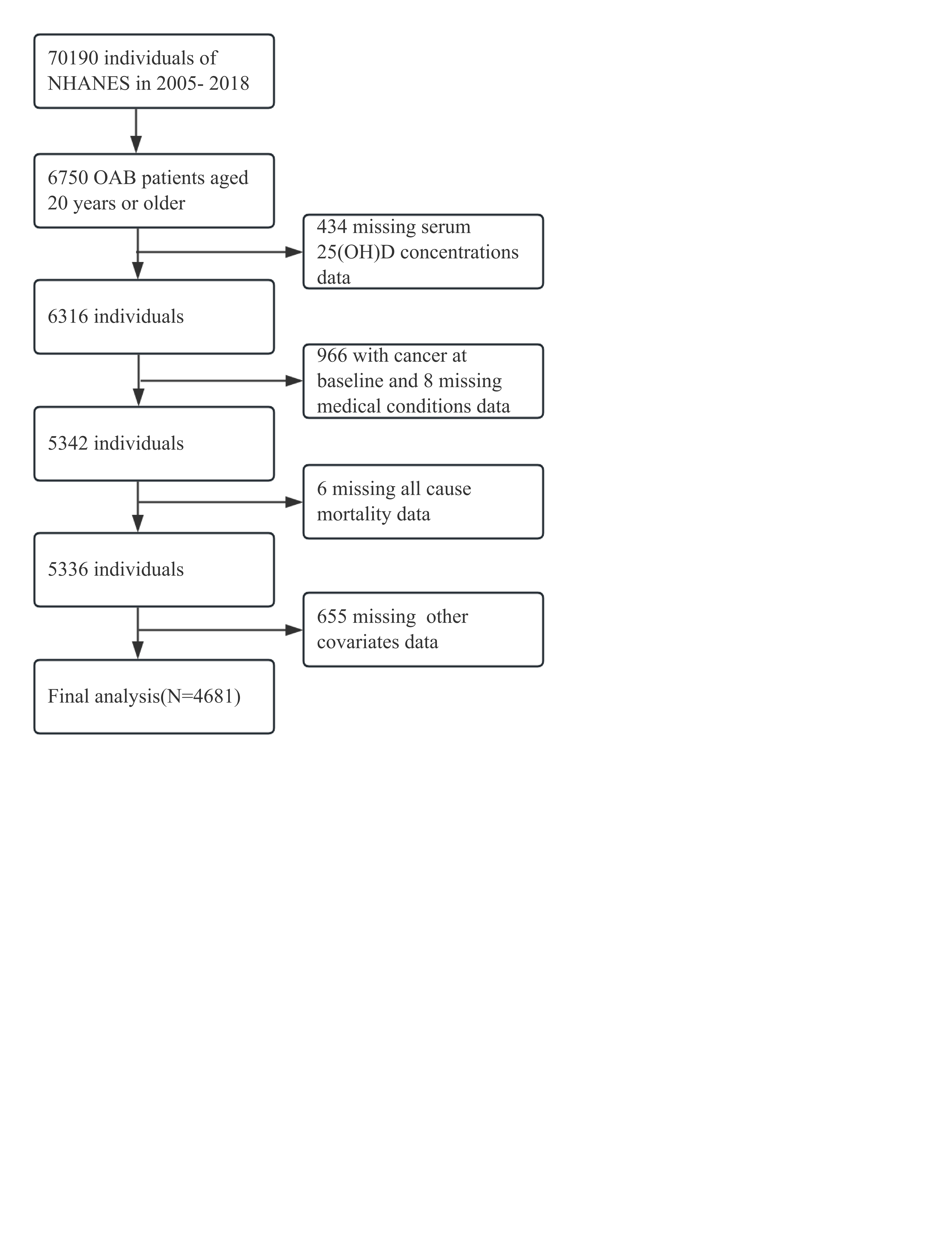

Supplement: Supplementary file 1 — Figure S1: Flowchart of participant screening. [file FSN3-14-e71722-s003.png]

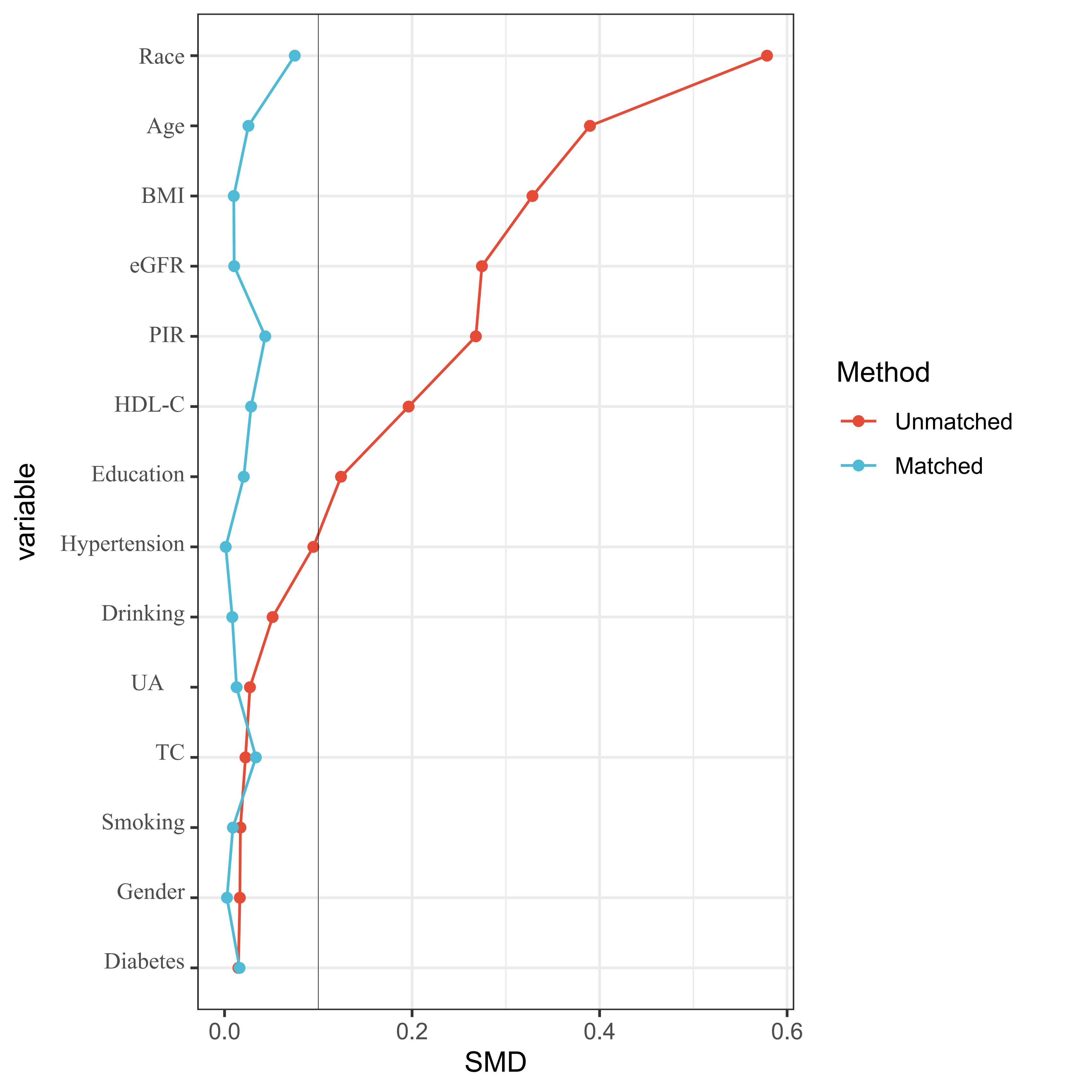

Supplement: Supplementary file 2 — Figure S2: Propensity score matching in two groups of vitamin D concentrations. [file FSN3-14-e71722-s001.png]
