# Supplementary material for: Association of Vitamin D Levels and Mortality in Overactive Bladder: Nonlinear Dose–Response and Threshold Effect
Source: Food Sci Nutr. 2026 Apr 2;14(4):e71722. doi: 10.1002/fsn3.71722 (PMC13045321; doi:10.1002/fsn3.71722)
Supplement: Supplementary file 3 — Table S1: Detailed description of the relevant variables of OAB in the NHANES database. Table S2: OAB symptom score from NHANES. Table S3: Sensitivity analysis. [file FSN3-14-e71722-s002.docx]

| S Table 1: Detailed description of the relevant variables of OAB in the NHANES database. | |
| --- | --- |
| Covariables | Description in NHANES |
| UUI | During the past 12 months, have you leaked or lost control of even a small amount of urine with an urge or pressure to urinate and you couldn't get to the toilet fast enough; |
| UUI | How frequently does this occur; |
| Nocturia | During the past 30 days, how many times per night did you most typically get up to urinate, from the time you went to bed at night until the time you got up in the morning; |

| S Table 2: OAB symptom score from NHANES | |
| --- | --- |
| **Urge urinary incontinence frequency** | **Urge urinary incontinence score** |
| Never | 0 |
| Less than once a month | 1 |
| A few times a month | 1 |
| A few times a week | 2 |
| Every day and/or night | 3 |
| **Nocturia frequency** | **Nocturia score** |
| 0 | 0 |
| 1 | 1 |
| 2 | 2 |
| 3 | 3 |
| 4 | 3 |
| 5 or more | 3 |

Note:

| S Table 3 Sensitivity analysis | | | | |
| --- | --- | --- | --- | --- |
|  | 25(OH)D<66.82(nmol/L) | 25(OH)D≥66.82(nmol/L) | HR (95%CI) | P-value |
|  | Deaths / Total | Deaths / Total |  |  |
| Excluding participants whose follow-up within 2 years. |  |  |  |  |
| Model 1 | 378/2499 | 237/1669 | 0.81 (0.68,0.96) | 0.014 |
| Model 2 | 378/2499 | 237/1669 | 0.8 (0.67,0.94) | 0.009 |
| Model 3 | 378/2499 | 237/1669 | 0.77 (0.65,0.91) | 0.003 |
| Excluding participants who had the extreme values |  |  |  |  |
| Model 1 | 452/2754 | 284/1880 | 0.77 (0.66,0.9) | 0.001 |
| Model 2 | 452/2754 | 284/1880 | 0.76 (0.65,0.88) | <0.001 |
| Model 3 | 452/2754 | 284/1880 | 0.73 (0.63,0.86) | <0.001 |
| Excluding participants with baseline CVD diseases |  |  |  |  |
| Model 1 | 279/2268 | 177/1483 | 0.87 (0.72,1.07) | 0.183 |
| Model 2 | 279/2268 | 177/1483 | 0.85 (0.69,1.04) | 0.107 |
| Model 3 | 279/2268 | 177/1483 | 0.82 (0.67,1.01) | 0.06 |
| PSM analysis | 339/1577 | 241/1577 | 0.74(0.63, 0.87) | <0.001 |

Model 1: Adjusted for age, gender, race, education, PIR;

Model 2: Adjusted for age, gender, race, education, PIR, BMI, diabetes, hypertension, smoking, drinking;

Model 3: Adjusted for age, gender, race, education, PIR, BMI, diabetes, hypertension, smoking, drinking,TC, direct HDL-C, UA, eGFR.

The covariates adjusted in Model 3 above were chosen to generate PSM.
